# Alloimmunisation to Donor Antigens and Immune Rejection Following Foetal Neural Grafts to the Brain in Patients with Huntington's Disease

**DOI:** 10.1371/journal.pone.0000166

**Published:** 2007-01-24

**Authors:** Pierre Krystkowiak, Véronique Gaura, Myriam Labalette, Amandine Rialland, Philippe Remy, Marc Peschanski, Anne-Catherine Bachoud-Lévi

**Affiliations:** 1 Department of Neurology and Movement Disorders, Equipe Associée 2683, Hospital R. Salengro, Regional University Hospital, Lille, France; 2 Centre National de la Recherche Scientifique/Commissariat à l'Energie Atomique Unité de Recherche Associée 2210, Service Hospitalier Frédéric Joliot, Orsay, France; 3 Service d'Immunologie, Faculté de Médecine et Centre Hospitalier Régional et Universitaire de Lille, Lille, France; 4 Assistance Publique/Hôpitaux de Paris, Service de Neurologie et Faculté de Médecine Paris 12, Centre Hospitalier Universitaire Henri-Mondor, Créteil, France; 5 Institut National de la Santé Et de la Recherche Médicale Unité 421, Faculté de Médecine de Créteil, Créteil, France; Laboratory of Neurogenetics, National Institutes of Health, United States of America

## Abstract

**Background:**

The brain is deemed “immunologically privileged” due to sparse professional antigen-presenting cells and lymphatic drainage, and to the blood-brain barrier. Although the actual extent of this privilege is controversial, there is general consensus about the limited need in intracerebral neural grafts for immunosuppressive regimens comparable to those used in other cases of allotransplantation. This has led over the past fifteen years to the use of either short-term or even no immunosuppression in most clinical trials with foetal neural transplant in patients with Parkinson's and Huntington's disease.

**Methodology/Principal Findings:**

We report biological demonstration of alloimmunisation without signs of rejection in four grafted patients out of 13 studied during the course of a clinical trial involving fetal neural transplantation in patients with Huntington's Disease. Biological, radiological and clinical demonstration of an ongoing rejection process was observed in a fifth transplanted patient. The rejection process was, however, fully reversible under immunosuppressive treatment and graft activity recovered within six months.

**Conclusions/Significance:**

There had been, up to date, no report of documented cases that could have cast a doubt on those procedures. Our results underline the need for a reconsideration of the extent of the so-called immune privilege of the brain and of the follow-up protocols of patients with intracerebral grafts. It also suggests that some of the results obtained in past studies with foetal neural transplants may have been biased by an unrecognized immune response to donor cells.

## Introduction

Transplantation of cells and tissues to the mammalian brain and CNS has been clinically assessed for more than 15 years, in several hundred patients with Parkinson or Huntington Disease. Because the brain was considered differently than almost all other organs, in terms of the possibility of an immune response, little consideration has been given up to now either to the potential existence of adverse consequences of a rejection process, or even to the existence any sign of allo-immunisation in patients. The mechanisms underlying this privilege are diverse, and probably cumulative, and results from the blood– brain barrier, the absence of professional antigen-presenting cells in the brain and the sparse lymphatic drainage from the central nervous system (reviewed in [1]). Experimental studies in animal models have, however, now revealed that the previously held view that the brain was an absolute immunologically privileged site allowing indefinite survival without rejection of grafts of cells was wrong. Activated lymphocytes can cross the BBB, certain cells such as microglia may have an antigen presenting cell capacity and there is lymphatic drainage from the brain into the cervical lymph nodes. Thus, the brain should be regarded as a site where immune responses can occur, albeit in a modified form. Up to now, however, there had been no report of an immune rejection process following foetal neural grafting to the brain of a patient. We report here a first human case of characterized rejection, and a number of transplanted patients with biological signs of all-immunisation against the antigens of the foetal donors.

## Material and Methods

A phase II study looking for therapeutic efficacy of foetal neural grafts to the brain of patients with Huntington's disease (“MIG-HD”, ref. NCT00190450) is currently ongoing in Europe, following the promising results of a pilot study [2], [3], [4]. The original protocol has been approved by the CCPPRB of the Hospital Henri-Mondor in Creteil on August 8, 2001. Additional protocols and modifications to the letter of information and informed consent related to the reported study were accepted by the same committee on May 17, 2005 and January 16, 2006. Specific information was given to patients, whose personal data are included in this paper and all signed and informed consent for publication.

This protocol involves a temporary immunosuppressive therapy with cyclosporin A stopped during the first year after bilateral grafting, azathioprin and prednisolone progressively decreased then stopped over the following months. The present study has been organized as an ancillary to the main trial. It concerned 13 patients (table 1), who had received foetal neural transplants in two successive stereotaxic neurosurgical sessions –first aiming at the right striatum, second at the left- three weeks to three months apart except in patient 2, in which a surgical complication (subdural hematoma) following the first graft precluded a second session.

**Table 1:**
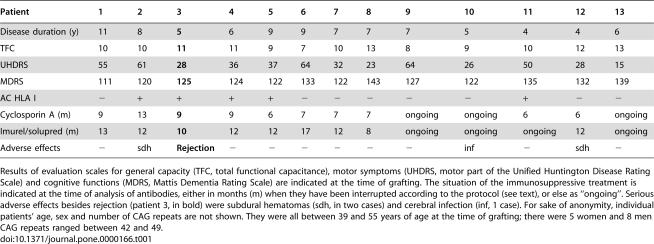
Clinical characteristics of the 13 grafted patients tested for the presence of antibodies against foetal donors' HLA I antigens.

Patient	1	2	3	4	5	6	7	8	9	10	11	12	13
Disease duration (y)	11	8	**5**	6	9	9	7	7	7	5	4	4	6
TFC	10	10	**11**	11	9	7	10	13	8	9	10	12	13
UHDRS	55	61	**28**	36	37	64	32	23	64	26	50	28	15
MDRS	111	120	**125**	124	122	133	122	143	127	122	135	132	139
AC HLA I	−	+	**+**	+	+	−	−	−	−	−	+	−	−
Cyclosporin A (m)	9	13	**9**	9	6	7	7	7	ongoing	ongoing	6	6	ongoing
Imurel/solupred (m)	13	12	**10**	12	12	17	12	8	ongoing	ongoing	ongoing	12	ongoing
Adverse effects	−	sdh	**Rejection**	−	−	−	−	−	−	inf	−	sdh	−

Results of evaluation scales for general capacity (TFC, total functional capacitance), motor symptoms (UHDRS, motor part of the Unified Huntington Disease Rating Scale) and cognitive functions (MDRS, Mattis Dementia Rating Scale) are indicated at the time of grafting. The situation of the immunosuppressive treatment is indicated at the time of analysis of antibodies, either in months (m) when they have been interrupted according to the protocol (see text), or else as “ongoing”. Serious adverse effects besides rejection (patient 3, in bold) were subdural hematomas (sdh, in two cases) and cerebral infection (inf, 1 case). For sake of anonymity, individual patients' age, sex and number of CAG repeats are not shown. They were all between 39 and 55 years of age at the time of grafting; there were 5 women and 8 men; CAG repeats ranged between 42 and 49.

Assessment protocols and imaging methods have been described in referenced studies [3], [4]. As a potential source for subsequent controls, patient's blood and foetal donor tissue were systematically taken and kept frozen, as part of the original protocol. Up to now, it had never been necessary to study those samples, which were defrozen for the present analysis in all 13 patients. Search for anti-HLA I and II antibodies against foetal donor antigens were not part of the original protocol (but are now an approved addition). They were carried out on blood samples, and in one case cerebrospinal fluid (patient 3), using classical techniques.

## Results

**Figure 1:**
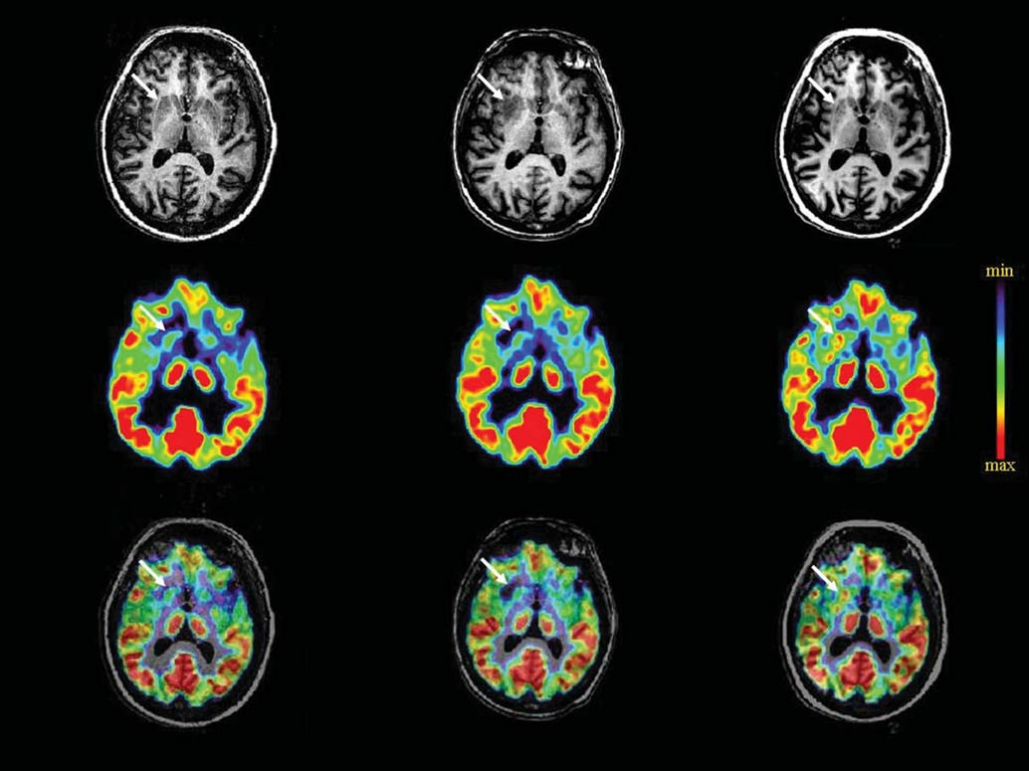
Brain imaging of the rejection process in patient 3 and its reversion under treatment. Magnetic resonance imaging and metabolic activity using ^18^F-deoxyglucose before surgery (T0), during the rejection process (T1) and after 6 months of reinstated immunosuppressive treatment (T2) are shown separately (upper and middle panel, respectively), then co-registered (lower panel). The white arrow indicates the right striatum. The false colour scale shows levels of metabolic activities from lowest (min) to highest (max).

Fourteen months after grafting, one patient (patient 3) started to exhibit general alteration of clinical status with weight loss of 22 kg over three months, worsened choreic movements lateralised to the left side, gait disorders and falls. Brain MRI showed an ongoing encephalitic process with vasogenic oedema, extending from the striatum to the frontal cortex mostly on the right side (Figure 1). Positron emission tomography with 18F-deoxyglucose revealed decreased metabolic activity in the same area of the right hemisphere (Figure 1), whereas striatal metabolism increased by about 50% on the left side, as commonly observed in the presence of functional grafts [3], [4].

After ruling out an infection on the basis of the lack of alteration of blood cells count, a graft rejection was hypothesized. A humoral response was indeed documented with anti-HLA I –but not II– antibodies in the blood (6 out of 30, i.e. cytotoxicity equivalent to 20% for a panel of 30 total lymphocyte suspensions) and in the cerebrospinal fluid (2 out of 30, i.e. cytotoxicity equivalent to 7% of the panel tested). The antibodies (IgG) were specifically directed against A3 and A11 class I HLAs. Control assay in a serum sample frozen immediately before surgery was negative for HLA antibodies, indicating post-operative occurrence of the specific B lymphocyte response. Biological demonstration of alloimmunisation against the graft tissues was provided by haplotyping preserved frozen samples from the three foetuses used for grafting, with HLA-A*3 allele in the foetus grafted on the right side, HLA-A*11 in one of the two foetuses grafted in the left striatum.

Aggressive acute anti-inflammatory treatment associated to reinstatement of the original immunosuppressive regimen rapidly led to regression of MRI signs of encephalitis. General clinical status remained altered for 4 more months then recovered, the patient regaining more than half of her lost weight. Anti-HLA I antibodies disappeared from blood and CSF samples. After 7 months of treatment, chorea lessened back to pre-rejection levels, while gait disorders and falls disappeared. The patient regained more than half of the lost weight. PET scan imaging then revealed the presence in the right striatum of two areas of increased metabolic activity (Figure 1) as compared not only to the hypometabolic state observed during the rejection episode, but also to preoperative values. After coregistration with the MRI, both areas suggest the presence of neural grafts.

Alloimmunisation to donors' antigens was sought in 12 other MIG-HD grafted patients followed up different times beyond the end of the period of immunosuppressive treatment. Four of them demonstrated alloimmunisation to one of the donors although none displayed either clinical or radiological signs of an ongoing rejection process (patients 2, 4, 5 and 11; Table 1). No specific pattern of alteration of blood cells counts could be identified in successive assessments over time that would differentiate the five alloimmunised patients (including the one with the ongoing rejection process) and the seven non-immunised patients (see Data S1). Because a search of alloimmunisation to foetal donor antigens was not part of the MIG-HD protocol before the identification of the rejection case reported here, it was not possible to determine the time of occurrence of HLA antibodies in the blood. It is interesting to mention, however, that one of the four positive patients (patient 11) had been transplanted less than six months before analysis as this shows that the humoral response to allo-antigens may occur under full immunosuppressive treatment, although it disappeared at a later time point, like in patient 3.

## Discussion

The main result of our study is the demonstration that a large proportion of patients who received intracerebral foetal neural grafts exhibited biological signs of allo-immunisation to donors' antigens, in general a few months after interruption of the immunosuppressive treatment. In one patient, this was accompanied by overt clinical, radiological and biological signs of an ongoing immune rejection process; this process was, however, fully reversible under treatment. These results call for a reconsideration of results of previous foetal neural grafts trials in which a deleterious effect of potentially similar immune responses was not examined. They underline the need for careful follow up of patients and appropriate immunosuppressive treatment in future clinical trials.

Our results show that foetal neural cells grafted to the brain may provoke a humoral response as other organ transplants [5], with specific alloimmunisation to donors' antigens in a significant proportion of patients. The role of the humoral response has been extensively studied in the pathogenesis of acute or hyperacute vascular rejection, but is now also demonstrated in chronic rejection. The appearance of HLA antibodies after transplantation is associated, in kidney, pancreas, heart and lung transplantation with poor transplant outcome and the occurrence of both acute and chronic rejection (review in [6]). After kidney transplantation, the humoral response was generally detected before transplant failure [7], suggesting that HLA antibodies could be responsible for transplant dysfunction. Previous studies have found wide variations in the percentage of patients with HLA antibodies after kidney transplantation, ranging between 12 and 60%. Our results are, thus, well in keeping with those obtained in other organ transplants, indicating that the immunological privilege of the brain may not extend as much to the activation of a humoral response. There are, however, major differences between the consequences of that allo-immunisation in the brain as compared to other organs. It is worth noting, first, that the presence of anti-HLA antibodies was not accompanied in all except one of our cases by overt signs of graft rejection. Second, even in the one case in whom an ongoing rejection process was identified, full reversion of the deleterious process could be obtained under treatment, including a functional recovery of the graft. This, together with previous evidence in animals [8], [9], showing in particular that triggering of an immune response may not lead to complete tissue rejection even in the presence of physiological signs, points to the existence of specific features of an immune response in the brain.

Our results call for reconsideration of the results of past clinical trials with foetal neural grafts performed, for most of them, in patients with Parkinson's disease. To our knowledge, there has been no systematic follow up of possible immune responses to grafts up to now in those trials, and immunosuppressive regimens have been extremely diverse, ranging from a classical long term tri-therapy [10], [11] down to no treatment altogether [12], with various intermediate situations comparable to the one used in this study [13,14]. What could have been the consequences of an inappropriate, or insufficiently maintained immunosuppressive regimen is but, at this point, a matter of speculation. Nevertheless, it is worth underlining the fact that at least in one large scale study a negative evolution of the results over time, following a first period of clinical improvement, was hypothetically related to the event of an otherwise undetermined immune response [14]. Also possibly relevant to our data is the appearance of major dyskinetic adverse effects in the long term following grafting in patients with Parkinson's disease [12]. Alloimmunisation to donors may indeed have contributed to those partially negative results and to the occurrence of delayed side-effects as, even in the absence of overt signs of a rejection process, effected neural grafts produce and elicit cytokine responses that may contribute to adverse functional effects observed in patients.

Our results clearly point to the fact that immune responses in the brain are complex, but cannot be ignored when it comes to repair strategies involving cellular transplants. New experimental studies are definitely needed in order to better comprehend and delineate the immunological privilege of the brain. The results of those experimental studies will allow us, in the future, to define more precisely the extent to which immunosuppressive regimens can be adapted to the specific case of intracerebral transplants. Until this redefinition is available, long term immunosuppressive treatment and thorough follow up of the immunological status of patients are likely indispensable.

## Supporting Information

Data S1 Blood cell counts. First grafting session is set at Time 0. Other times are indicated in months in relation to it. Counts corresponding to periods of presence of anti-foetal HLA antibodies identified in the blood of the patients are in bold.
(0.27 MB DOC)
