# Supplementary material for: Alloimmunisation to Donor Antigens and Immune Rejection Following Foetal Neural Grafts to the Brain in Patients with Huntington's Disease
Source: PLoS One. 2007 Jan 24;2(1):e166. doi: 10.1371/journal.pone.0000166 (PMC1764859; doi:10.1371/journal.pone.0000166)
Supplement: Data S1 — Blood cell counts. First grafting session is set at Time 0. Other times are indicated in months in relation to it. Counts corresponding to periods of presence of anti-foetal HLA antibodies identified in the blood of the patients are in bold. (0.27 MB DOC) [file pone.0000166.s001.doc]

# Supplementary data

**Blood cell counts.** First grafting session is set at Time 0. Other times are indicated in months in relation to it. Counts corresponding to periods of presence of anti-fœtal HLA antibodies identified in the blood of the patients are in bold.

| **1** | -1 | 0 | +1 | +2 | +3 | +4 | +13 | +19 | +20 |
| --- | --- | --- | --- | --- | --- | --- | --- | --- | --- |
| Leucocytes10^9^/l | 6.7 | 5.3 | 6,6 | 6.3 | 10,6 | 7,4 | 6,9 | 6.9 | 9,1 |
| Red blood cells10^12^/l | 4.7 | 4.8 | 4,1 | 4.2 | 3,7 | 4,2 | 4,5 | 4.7 | 4,3 |
| Haemoglobin g/dl | 14.4 | 14.9 | 12,7 | 13.4 | 11, 9 | 13,4 | 14 | 14.7 | 13,6 |
| Haematocrit % | 43 | 44 | 38 | 39 | 35 | 41 | 43 | 43 | 40 |
| Platelets 10^9^/l | 262 | 238 | 212 | 263 | 210 | 254 | 339 | 248 | 233 |
| Neutro % | 68 | 55 | 45 | 63 | 68 | 65 | 67 | 66 | 78 |
| Eosino % | 1 | 2 | 2 | 0 | 1 | 1 | 1 | 1 | 0 |
| Baso % | 0 | 0 | 0 | 0 | 0 | 0 | 1 | 0 | 0 |
| Lymphocytes % | 26 | 37 | 47 | 30 | 23 | 29 | 26 | 27 | 17 |
| Monocytes % | 5 | 6 | 7 | 7 | 8 | 5 | 6 | 6 | 6 |

| **2** | -3 | -1 | +2 | +3 | +6 | +12 | +18 | **+24** | **+32** |
| --- | --- | --- | --- | --- | --- | --- | --- | --- | --- |
| Leucocytes10^9^/l | 6.3 | 5.5 | 7 | 7 | 7.8 | 4.8 | 5.8 | **9.4** | **5.9** |
| Red blood cells10^12^/l | 4.4 | 4.3 | 4.1 | 4.2 | 4.2 | 3.7 | 4.3 | **4.6** | **4.5** |
| Haemoglobin g/dl | 13 | 13 | 11.9 | 12.8 | 12.9 | 11.6 | 13 | **13.3** | **13.5** |
| Haematocrit % | 45 | 38 | 36 | 37 | 37 | 34 | 38 | **40** | **39** |
| Platelets 10^9^/l | 253 | 210 | 302 | 235 | 253 | 227 | 233 | **277** | **259** |
| Neutro % | 58 | 54 | 56 | 56 | 52 | 58 | 58 | **73** | **56** |
| Eosino % | 4 | 4 | 3 | 2 | 2 | 2 | 3 | **1** | **2** |
| Baso % | 1 | 1 | 1 | 1 | 0 | 1 | 1 | **0** | **1** |
| Lymphocytes % | 32 | 35 | 34 | 37 | 41 | 34 | 33 | **22** | **35** |
| Monocytes % | 5 | 6 | 6 | 4 | 5 | 5 | 6 | **4** | **6** |

| **3** | 0 | +3 | +4 | +6 | **+12** | +18 | +24 |
| --- | --- | --- | --- | --- | --- | --- | --- |
| Leucocytes10^9^/l | 5.4 | 5.7 | 6.3 | 5.7 | **4.2** | 8.3 | 6.3 |
| Red blood cells10^12^/l | 4.29 | 4.4 | 4.79 | 4.40 | **4.10** | 4.65 | 4.41 |
| Haemoglobin g/dl | 13.1 | 14.1 | 13.7 | 14.1 | **12.5** | 14.8 | 13.8 |
| Haematocrit % | 32.8 | 41.7 | 41.2 | 41.7 | **37.4** | 44.1 | 42 |
| Platelets 10^9^/l | 130 | 250 | 191 | 250 | **213** | 183 | 174 |
| Neutro % | 63 | 66 | 66 | 66 | **59** | 68 | 54 |
| Eosino % | 2 | 2 | 1 | 2 | **6** | 1 | 6 |
| Baso % | 0 | 0 | 0 | 0 | **0** | 0 | 0 |
| Lymphocytes % | 28 | 27 | 26 | 27 | **28** | 25 | 33 |
| Monocytes % | 7 | 5 | 7 | 5 | **7** | 6 | 7 |

| **4** | -1 | 0 | +1 | +3 | +3 | +3 | +5 | +12 | +18 | +18 | **+24 (2 sets)** | **+26** |
| --- | --- | --- | --- | --- | --- | --- | --- | --- | --- | --- | --- | --- |
| Leucocytes10^9^/l | 5.6 | 5.6 | 10.7 | 6.4 | 14.1 | 8.4 | 8 | 7.3 | 5.9 | 6.9 | **6.1 (6.7)** | **8.3** |
| Red blood cells10^12^/l | 4.6 | 4.8 | 4.87 | 4.4 | 4 | 4.5 | 4.6 | 4.3 | 4.3 | 4.7 | **4.8 (4.7)** | **4.7** |
| Haemoglobin g/dl | 14.3 | 14.8 | 14.8 | 14 | 12.9 | 14.5 | 15 | 14.2 | 14.1 | 15.6 | **15.2 (15.1)** | **15.4** |
| Haematocrit % | 42 | 43 | 45 | 41 | 32 | 42 | 43 | 41 | 42 | 46 | **46 (44)** | **45** |
| Platelets 10^9^/l | 245 | 293 | 322 | 277 | 258 | 320 | 305 | 260 | 244 | 294 | **257 (251)** | **263** |
| Neutro % | 57 | 53 | 54 | 52 | ND | 58 | 58 | 70 | 63 | 67 | **65 (66)** | **68** |
| Eosino % | 3 | 2 | 1 | 2 | ND | 2 | 1 | 2 | 1 | 1 | **1 (1)** | **0** |
| Baso % | 1 | 1 | 1 | 0 | ND | 1 | 0 | 0 | 0 | 1 | **0 (0)** | **0** |
| Lymphocytes % | 31 | 37 | 38 | 38 | ND | 32 | 33 | 18 | 27 | 22 | **27 (27)** | **21** |
| Monocytes % | 8 | 7 | 6 | 9 | ND | 8 | 8 | 11 | 9 | 9 | **7 (7)** | **10** |

| **5** | -3 | +6 | +9 | **+13** | **+19** |
| --- | --- | --- | --- | --- | --- |
| Leucocytes10^9^/l | 4.6 | 6.4 | 7.7 | **7** | **7.7** |
| Red blood cells10^12^/l | 4.38 | 4.02 | 3.57 | **4.31** | **4.40** |
| Haemoglobin g/dl | 13.3 | 12.7 | 11.2 | **13** | **13.4** |
| Haematocrit % | 39.2 | 37.5 | 32.7 | **39.4** | **39.5** |
| Platelets 10^9^/l | 271 | 307 | 300 | **278** | **283** |
| Neutro % | 57 | 45.9 | 53.7 | **55.7** | **55.8** |
| Eosino % | 1.7 | 1.2 | 1.4 | **1.1** | **0.9** |
| Baso % | 0.9 | 0.5 | 0.3 | **0.4** | **0.3** |
| Lymphocytes % | 30.8 | 41.2 | 34.7 | **34.6** | **34.6** |
| Monocytes % | 9.6 | 11.2 | 9.9 | **8.2** | **8.4** |

| **6** | -24 | -2 | 0 | 0 | 0 | +1 | +1 | +1 | +6 | +12 |
| --- | --- | --- | --- | --- | --- | --- | --- | --- | --- | --- |
| Leucocytes10^9^/l | 4.9 | 3.9 | 4.2 | 12.3 | 9.3 | 6.8 | 14.4 | 6.3 | 7.1 | 5.7 |
| Red blood cells10^12^/l | 4.1 | 4.5 | 4.5 | 3.8 | 4.4 | 4.1 | 3.8 | 3.5 | 4.2 | 4.6 |
| Haemoglobin g/dl | 11.4 | 13 | 12.8 | 11.2 | 12.6 | 11.8 | 11 | 10.2 | 11.6 | 12.3 |
| Haematocrit % | 35 | 38 | 37 | 32 | 37 | 34 | 31 | 30 | 35 | 38 |
| Platelets 10^9^/l | 253 | 239 | 242 | 202 | 235 | 219 | 218 | 185 | 270 | 272 |
| Neutro % | 66 | 59 | 55 | 90 | 66 | 56 | 88 | 55 | 75 | 54 |
| Eosino % | 4 | 3 | 2 | 0 | 1 | 1 | 0 | 1 | 2 | 3 |
| Baso % | 1 | 1 | 0 | 0 | 0 | 1 | 0 | 0 | 1 | 1 |
| Lymphocytes % | 25 | 32 | 38 | 7 | 27 | 37 | 8 | 39 | 19 | 34 |
| Monocytes % | 4 | 5 | 5 | 3 | 5 | 5 | 3 | 5 | 4 | 8 |

| **7** | -1 | 0 | +1 | +2 | +3 | +12 | +18 |
| --- | --- | --- | --- | --- | --- | --- | --- |
| leucocytes10^9^/l | 5.1 | 4.3 | 5.7 | 7.0 | 5.7 | 4.3 | 5.6 |
| Red blood cells10^12^/l | 4.9 | 4.7 | 4.5 | 4.2 | 4.3 | 4.5 | 4.5 |
| Haemoglobin g/dl | 14.9 | 14.3 | 13.9 | 12.9 | 13.2 | 13.8 | 13.9 |
| Haematocrit % | 42 | 41 | 40 | 37 | 38 | 40 | 40 |
| Platelets 10^9^/l | 228 | 202 | 255 | 249 | 235 | 249 | 244 |
| Neutro % | 58 | 54 | 49 | 64 | 62 | 68 | 61 |
| Eosino % | 1 | 1 | 1 | 1 | 1 | 1 | 1 |
| Baso % | 1 | 0 | 1 | 1 | 1 | 1 | 1 |
| Lymphocytes % | 35 | 40 | 43 | 28 | 30 | 25 | 32 |
| Monocytes % | 6 | 5 | 6 | 7 | 7 | 5 | 6 |

| **8** | -3 | 0 | 0 | 0 | +1 | +1 | +1 | +6 |
| --- | --- | --- | --- | --- | --- | --- | --- | --- |
| Leucocytes10^9^/l | 6.1 | 5.9 | 16.9 | 8.7 | 7.4 | 8.9 | 8.2 | 10.1 |
| Red blood cells10^12^/l | 4.8 | 4.6 | 4.5 | 4.7 | 4.4 | 4.0 | 4.5 | 4.7 |
| Haemoglobin g/dl | 14.8 | 14 | 14.1 | 14.4 | 13.4 | 12.5 | 13.9 | 14.9 |
| Haematocrit % | 43 | 41 | 41 | 41 | 40 | 36 | 40 | 43.5 |
| Platelets 10^9^/l | 287 | 258 | 253 | 269 | 282 | 244 | 293 | 291 |
| Neutro % | 47 | 41 | 84 | 49 | 43 | 54 | 46 | 50 |
| Eosino % | 1 | 3 | 0 | 2 | 3 | 0 | 3 | 2 |
| Baso % | 1 | 0 | 0 | 0 | 1 | 0 | 0 | 1 |
| Lymphocytes % | 42 | 47 | 11 | 43 | 45 | 30 | 43 | 37 |
| Monocytes % | 9 | 8 | 5 | 6 | 9 | 8 | 9 | 8 |

| **9** | -3 | 0 | +2 | +3 | +4 | +6 | +10 |
| --- | --- | --- | --- | --- | --- | --- | --- |
| Leucocytes10^9^/l | 6.1 | 7.7 | 5 | 7.2 | 4.0 | 5.0 | 10 |
| Red blood cells10^12^/l | 4.3 | 4 | 3.9 | 3.8 | 3.5 | 3.9 | 3.4 |
| Haemoglobin g/dl | 13.7 | 12.6 | 12.1 | 12.2 | 11.2 | 12.4 | 11.1 |
| Haematocrit % | 40 | 38 | 37 | 35 | 33 | 37 | 32 |
| Platelets 10^9^/l | 232 | 223 | 226 | 226 | 206 | 219 | 245 |
| Neutro % | 65 | ND | ND | ND | 50 | 51 | 71 |
| Eosino % | 2 | ND | ND | ND | 2 | 1 | 1 |
| Baso % | 1 | ND | ND | ND | 0 | 1 | 0 |
| Lymphocytes % | 26 | ND | ND | ND | 38 | 37 | 22 |
| Monocytes % | 7 | ND | ND | ND | 10 | 11 | 7 |

| **10** | -2 | +5 | +8 | +19 |
| --- | --- | --- | --- | --- |
| Leucocytes10^9^/l | 7.3 | 7.1 | 7.2 | 5.3 |
| Red blood cells10^12^/l | 4.7 | 4.7 | 4.6 | 5 |
| Haemoglobin g/dl | 14.1 | 14 | 14 | 15.6 |
| Haematocrit % | 42.5 | 41.5 | 41.8 | 44.9 |
| Platelets 10^9^/l | 227 | 261 | 224 | 237 |
| Neutro % | 70 | 60 | 56 | 53 |
| Eosino % | 6 | 4 | 2 |  |
| Baso % | 0 | 1 | 0 | 1 |
| Lymphocytes % | 13 | 29 | 34 | 38 |
| Monocytes % | 11 | 6 | 8 | 8 |

| **11** | 0 | +3 | **+4** | +6 |
| --- | --- | --- | --- | --- |
| Leucocytes10^9^/l | 5.6 | 6.1 | **6.3** | 5.6 |
| Red blood cells10^12^/l | 4.65 | 4.41 | **4.79** | 4.40 |
| Haemoglobin g/dl | 13.70 | 12.8 | **13.7** | 12.6 |
| Haematocrit % | 40.4 | 37.7 | **41.2** | 37.1 |
| Platelets 10^9^/l | 246 | 182 | **191** | 182 |
| Neutro % | 59 | 64 | **66** | 58 |
| Eosino % | 2 | 2 | **1** | 1 |
| Baso % | 0 | 0 | **0** | 0 |
| Lymphocytes % | 31 | 27 | **26** | 34 |
| Monocytes % | 8 | 7 | **7** | 7 |

| **12** | -1 | +8 | +9 |
| --- | --- | --- | --- |
| Leucocytes10^9^/l | 4.8 | 7.9 | 7.7 |
| Red blood cells10^12^/l | 4.42 | 4.14 | 3.57 |
| Haemoglobin g/dl | 13.4 | 12.4 | 11.2 |
| Haematocrit % | 39 | 36 | 33 |
| Platelets 10^9^/l | 180 | 204 | 300 |
| Neutro % | 68 | 80 | 54 |
| Eosino % | 3 | 1 | 1 |
| Baso % | 0 | 0 | 0 |
| Lymphocytes % | 22 | 14 | 35 |
| Monocytes % | 7 | 5 | 10 |

| **13** | -1 | 0 | +1 | +1 | +2 | +3 |
| --- | --- | --- | --- | --- | --- | --- |
| Leucocytes10^9^/l | 5.4 | 4.9 | 7.0 | 7.5 | 6.6 | 5.9 |
| Red blood cells10^12^/l | 4.4 | 4.3 | 4.3 | 4.2 | 4.5 | 4.4 |
| Haemoglobin g/dl | 14 | 14 | 14.3 | 13.7 | 14.5 | 14.6 |
| Haematocrit % | 42 | 42 | 42 | 41 | 43 | 43 |
| Platelets 10^9^/l | 274 | 248 | 276 | 243 | 292 | 316 |
| Neutro % | 63 | 61 | 57 | 73 | 56 | 77 |
| Eosino % | 1 | 2 | 1 | 1 | 2 | 1 |
| Baso % | 1 | 1 | 1 | 0 | 1 | 0 |
| Lymphocytes % | 24 | 25 | 28 | 15 | 30 | 13 |
| Monocytes % | 11 | 11 | 13 | 11 | 11 | 9 |
